# Assessment of prenatal exposure to tobacco smoke by cotinine in cord blood for the evaluation of smoking control policies in Spain

**DOI:** 10.1186/1471-2393-12-26

**Published:** 2012-04-05

**Authors:** Carme Puig, Oriol Vall, Óscar García-Algar, Esther Papaseit, Simona Pichini, Esteve Saltó, Joan R Villalbí

**Affiliations:** 1 Unitat de Recerca i Entorn (URIE), Institut Municipal d'Investigacions Mèdiques (IMIM)-Hospital del Mar, Parc de Salut Mar, Barcelona, Spain; 2 Department of Pediatrics, Universitat Autònoma de Barcelona (UAB), Barcelona, Spain; 3 Red Salud Materno Infantil y del Desarrollo (SAMID), RETIC Instituto Carlos III, Madrid, Spain; 4 Unitat de Recerca en Farmacologia (URF), Institut Municipal d'Investigacions Mèdiques (IMIM)-Hospital del Mar, Parc de Salut Mar, Barcelona, Spain; 5 Department of Therapeutic Research and Medicines Evaluation, Istituto Superiore di Sanità, Rome, Italy; 6 Unitat de Recerca i Control del Tabaquisme, Institut Català d'Oncologia-IDIBELL, L'Hospitalet de Llobregat, Spain; 7 Department of Public Health, Generalitat de Catalunya, Barcelona, Spain; 8 Agència de Salut Pública de Barcelona, Barcelona, Spain; 9 Institute of Biomedical Research (IIB Sant Pau), Barcelona, Spain; 10 CIBER ESP, Barcelona, Spain; 11 Hospital del Mar, Pg. Marítim 25-29, 08003 Barcelona, Spain

## Abstract

**Background:**

Over the last few years a decreasing trend in smoking has occurred not only in the general population but also during pregnancy. Several countries have implemented laws requiring all enclosed workplace and public places to be free of second hand smoke (SHS). In Spain, legislation to reduce SHS was implemented in 2005. The present study examines the possible effect of this legislation on prenatal SHS exposure.

**Methods:**

Mothers and newborns were recruited from 3 independent studies performed in Hospital del Mar (Barcelona) and approved by the local Ethics Committee: 415 participated in a study in 1996-1998, 283 in 2002-2004 and 207 in 2008. A standard questionnaire, including neonatal and sociodemographic variables,tobacco use and exposure during pregnancy, was completed at delivery for all the participants in the three study groups. Fetal exposure to tobacco was studied by measuring cotinine in cord blood by radioimmunoassay (RIA).

**Results:**

32.8% of the pregnant women reported to smoke during pregnancy in 1996-1998, 25.9% in 2002-2004 and 34.1% in 2008. In the most recent group, the percentage of no prenatal SHS exposure (cord blood cotinine 0.2-1 ng/mL) showed an increase compared to the previous groups while the percentages of both: low (1.1-14 ng/mL) and very high (> 100 ng/mL) prenatal SHS exposure showed a decrease.

**Discussion:**

The results of the three study periods (1996-2008) demonstrated a significant increase in the percentage of newborns free from SHS exposure and a decrease in the percentage of newborns exposed to SHS during pregnancy, especially at the very high levels of exposure. A significant maternal smoking habit was noted in this geographical area with particular emphasis on immigrant pregnant smoking women.

**Conclusions:**

Our study indicates that there is a significant maternal smoking habit in this geographical area. Our recommendation is that campaigns against smoking should be directed more specifically towards pregnant women with particular emphasis on non-native pregnant smokers due to the highest prevalence of tobacco consumption in the immigrant women.

## Background

Exposure to second hand smoke (SHS), active or passive, is considered the single most important cause of avoidable morbidity and early mortality in many industrialized countries [1]. Over the last years, Spain presented a decreasing trend in smoking not only in the general population but also during pregnancy [2-5]; however it is still an important risk factor for infant health. Most recent population data from Barcelona documented that 28% of pregnant women smoke at the beginning of pregnancy, and although 42% of them quit during the gestational period, 16% smoke throughout pregnancy [5] therefore the gestational and fetal effects of cigarette smoke are of great importance for public health [6]. The effects of tobacco smoke exposure on the health of pregnant women are associated with an increased risk of spontaneous abortion, low birth weight, prematurity, perinatal death and sudden infant death syndrome [7-9]. while the risk of cognitive problems and neurodevelopment growth [10], childhood cancer [11] and respiratory and allergic symptomatology in the first years of life [12] can be increased as well.

There is scientific evidence that smoke-free environment is the only strategy protecting the population from the negative effects of SHS [13]. For this reason, several countries have implemented legislations requiring all enclosed workplace and public places to be free of SHS [14]. Ireland was the first country with comprehensive smoke-free legislation implemented in 2004. Since then, countries like Norway, New Zealand, Italy, Uruguay, England and many provinces or states in Canada, the USA or Australia [15,16] followed. Recently, by measuring air nicotine concentrations, a study had shown that exposure to second hand smoke has decreased greatly in indoor public places and workplaces in Uruguay after the implementation of a comprehensive national smoke-free legislation [17].

Spain introduced the legislation to reduce SHS in 2005. The Law 28/2005 includes a health recommendations against smoking and recommendations forregulation of tobacco smoking in public places with wide exemptions in bars, restaurants and night clubs [18,19]. Furthermore, the implementation of smoke-free legislation generated a continuous discussion topic in the media [20]. After the implementation of the Spanish smoke-free legislation [3,4,21-23], programs to help smoking pregnant women to quit were implemented (program "Embaràs fum") in Catalonia. These programs included specific training and free nicotine replacement treatment for pregnant smoking women throughout pregnancy [24].

Prenatal tobacco exposure has been usually assessed using self-reported maternal questionnaire [25-28]. Difficulties in differentiating passive tobacco exposure from active smoking (such as reluctance to admit active smoking or being unconscious of passive exposure) have prompted the use of specific biomarkers to prevent bias in self-reporting questionnaires [29,30]. Nicotine and its main metabolite (cotinine) have been used as biomarkers for SHS in conventional (blood and urine) [31,32] and non-conventional matrices (saliva, meconium and hair) [33,34]. Cotinine presents a longer biological half-life than nicotine and it is considered the best biochemical marker to differentiate between active and passive tobacco exposure [33,35-37]. Moreover, the levels of cotinine have been found to be directly related to daily cigarette consumption [35]. Cotinine cord blood is the most sensitive and least invasive measure of prenatal SHS in newborns [36-42].

The present study examines the possible effects of the actions taken to prevent prenatal SHS exposure. The relationship between the implementation of the law against smoking and a decrease in maternal tobacco use and exposure during pregnancy has been studied by measuring cotinine in cord blood as a reliable biomarker of active and/or passive maternal exposure to SHS. In addition, the relationship between the newborn's cotinine level in cord blood and the parents' smoking pattern declared by questionnaire has been also evaluated.

## Methods

### Design and subjects

The study was carried out on non selected (general population) mothers and newborns from 3 independent studies conducted at different times in the same setting: Hospital del Mar in Barcelona (Catalonia, Spain

#### Study 1

415 mothers and their infants were recruited during the period 1996-98, from the Asthma Multicenter Infant Cohort Study (AMICS). The AMICS study was designed to investigate the effects of several pre and postnatal environmental exposures on the inception of atopy and asthma [12,29,41,43].

#### Study 2

283 mothers and their infants were recruited from 2002 until 2004, before the general implementation of smoke-free workplace policy in Spain in 2005, from the MECONIUM-1 study. The MECONIUM-1 study aimed to estimate the prevalence of using drugs of abuse by pregnant women and the subsequent foetal exposure to illicit drugs by testing the meconium of the newborn [44].

#### Study 3

207 mothers and their infants were recruited during 2008, after the implementation of smoke-free workplace policy in Spain, from MECONIUM-2 study. The MECONIUM-2 study aimed to estimate the prevalence of alcohol use by pregnant women and the subsequent foetal exposure to alcohol using meconium testing [45].

Informed consent was obtained from parents and all three studies were previously approved by the local Ethics Committee (Comitè d'Ètica i Investigació Clínica, Institut Municipal d'Assistència Sanitària; 2006/2831/I-extension).

### Questionnaire information

The same standard questionnaire previously reported in the literature [12,29,33,37,41] was completed at delivery for all the participants, regardless of the group they belonged to. The questionnaire was always administered by the study interviewer immediately after postpartum and included sociodemographic characteristics (parents' age, parents' birth country, social class defined by parents' occupation using the UK Registrar General's 1990 classification which categorizes people with similar levels of occupational skills). In addition, mothers were asked about their smoking habit and passive exposure to tobacco smoke during pregnancy. If they were smokers, the average number of cigarettes smoked during pregnancy was recorded and the nicotine daily intake (NDI), obtained from the average number of cigarettes smoked per day multiplied by nicotine content (in milligrams) of each cigarette, was calculated. Regarding exposure to SHS, mothers were asked if they were regularly exposed to SHS at home and by whom (nobody smoked, the father or the mother was the only smoker or both smoked). The gender of the newborn was also recorded.

### Cotinine in cord blood

Umbilical cord blood was obtained at the time of delivery and immediately centrifuged. Serum was collected and stored at -80°C until analysis. Cotinine was analysed by RIA. The detectable range of measurement from the standard curve was 0.2-20 ng/mL cotinine, with an interassay coefficient of variation of 6-10%. A cut-off value of 14 ng/mL (previously proposed by different authors) was used for cord serum cotinine to discern between newborns of non-smoking mothers from smoking mothers [30]. Newborns exposed to SHS were categorised according to cord blood cotinine level as non exposed (cord serum cotinine < 1 ng/mL), low exposure (cord serum cotinine from 1 to 14 ng/mL), medium exposure (cord serum cotinine from 14 to 50 ng/mL), high exposure (cord serum cotinine > 50 ng/mL) and very high exposure (cord serum cotinine > 100 ng/mL) [30,37].

### Statistical methods

We compared sociodemographic data of different study groups using the Chi-square test or Fisher's exact test for categorical variables and Student's t-test for continuous variables. Furthermore, to compare parental smoking habits among each period of the study a logistic regression analysis with odds ratio (OR) was conducted. To assess the relationship between parental smoking habit and the mean cord blood cotinine levels, a univariate statistical analysis was used with cotinine levels as continuous variable and parental smoking habit as random factor. The categories of prenatal exposure were assessed by logistic multinomial regression. The independent variables were adjusted for socioeconomic status, mother and father's nationalities, mother's age and newborn's sex. Differences associated with p values lower than 0.05 were considered statistically significant. The SPSS program (version 16.0, SPSS Inc, Chicago, IL, USA) was used for the analysis of data.

## Results

Table 1 shows the socioeconomics characteristics of the study groups. The differences between the 3 periods studied reflect the sociodemographic changes in the population attending at the Hospital del Mar. Recently, the percentage of foreign immigrants has increased significantly (practically doubled); the percentage of professionals has also grown as a result of a higher number of middle class people being attracted by the sea-side life in Barcelona.

**Table 1:** Sociodemographic characteristics of the study population

	1996 - 98 N = 415	2002 - 04 N = 283	2008 N = 207
**Mother's age**, mean (SD)	29.1 (5.4)	29.0 (6.1)	30.6 (6.2)
**Father's age**, mean (SD)	32.0 (6.0)	NA	33.3 (7.0)
**Mother's nationality **(Spain), %	74.5	51.3**	46.6
**Father's nationality **(Spain), %	74.8	51.3**	44.6
**Mother's working **(yes), %	51.8	52.8	61.9
**Father's working **(yes), %	91.8	91.4	95.4
**Mother's profession**, %	6.9	10.8*	14.6†
Professional	17.5	12.4	12.5
Managerial and technical	37.6	35.8	41.6
Skilled (non-manual)	21.7	24.5	18.8
Skilled (manual)	16.4	16.5	12.5
Unskilled			
**Father's profession**, %	6.2	8.9	16.7†
Professional	10.7	10.9	6.9
Managerial and technical	22.7	19.4	12.5
Skilled (non-manual)	57.8	56.6	58.3
Skilled (manual)	2.6	4.2	5.6
Unskilled			

NA: not available

* p-value in relation to 1996-98; p-value < 0.05

** p-value in relation to 1996-98; p-value < 0.001

† p-value in relation to 2002-04; p-value < 0.05

### Parents' smoking habit

According to the self-reported questionnaires, 32.8% of the pregnant women reported daily smoking during pregnancy in 1996-98, 25.9% in 2002-04 and 34.1% in 2008; the statistics don't show significant differences in the number of mothers who smoked throughout pregnancy over the 12 years of the study. There were no significant differences between the groups regarding the self-reported tobacco use if maternal smoking expressed as NDI among the 3 groups of study is compared: mean = 5.60 (range: 0.80-28.00) in 1996-98, mean = 7.50 (range: 0.70-32.00) in 2002-04 and mean = 10.50 (range: 1.20-32.00) in 2008.

Assessing how smoking habit in families has varied with respect to mother's country of origin, a reduction in native homes where only the father was smoking: 29.6% in 1996-1998 and 13.7% in 2002-2004 (OR: 0.46; CI 95%: 0.23-0.95) was observed; however, this trend was not maintained in 2008 (25.7%). In homes where only the mother was smoking, there was an increase from 1996-1998 (11.5%) to 2002-2004 (25.5%) (OR: 2.31; CI 95%: 1.18-4.52) and although this trend was broken in 2008 (14.3%), it was without statistical significance. There was no significant change in homes with both parents smoking. In immigrant homes, there were no differences in the three study periods when only the father (34.1%, 22.4% and 23.1%) or only the mother (4.5%, 7.1% and 10.3%) were smoking; the number of homes where both parents were smoking was 3.4% in 1996-1998, 5.9% in 2002-2004, and increased to 23.1% in 2008 (OR: 5.82; CI 95%: 1.72-19.75).

### Cotinine as biomarker of fetal SHS

The mean cord blood cotinine levels in the newborns from all the study groups were: 3.21 (range: 0.20-910.00) in 1996-98, 0.80 (range: 0.20-250.00) in 2002-04 and 0.44 (range: 0.20-128.00) in 2008.

Table 2 shows the stratification of prenatal SHS exposure based on cord blood cotinine in the five categories defined previously. The stratification criteria were the same as in a previous publication [37]. The results show an increase in the percentage of no SHS exposure in the most recent group compared to the previous groups while the percentages of both prenatal SHS exposure categories: low (1.1-14 ng/mL) and very high (> 100 ng/mL) show a decrease.

**Table 2:** Prenatal SHS exposure by measuring cotinine in cord blood

		1996 - 98	2002 - 04		2008		
		N = 415	N = 283		N = 207		
**Level of prenatal SHS (cotinine ng/mL)**		**%**	**% OR (CI 95%)**	**OR Adjust (CI 95%) %**		**OR (CI 95%)**	**OR Adjust (CI 95%)**

Non exposure	< 1	10.8	56.9 1.18 (1.11-1.26)	** 1.14 (1.06-1.22)	** 73.4 1.20 (0.97-1.48)		1.22 (0.96-1.55)
Low exposure	1-14	54.9	19.8 0.88 (0.82-0.93)	** 0.86 (0.80-0.92)	** 12.1 0.93 (0.73-1.20)		0.99 (0.75-1.31)
Medium exposure	14-50	9.4	11.0 1	1	8.2	1	1
High exposure	50-100	7.5	8.8 1.00 (0.93-1.08)	1.01 (0.93-1.10)	3.9	0.84 (0.60-1.16)	0.83 (0.56-1.23)
Very high exposure	> 100	17.3	3.5 0.82 (0.75-0.90)	** 0.83 (0.76-0.91)	** 2.4	0.97 (0.64-1.46)	0.99 (0.64-1.55)

Adjust: adjusted by gender of newborn, age and mother's nationality

* p-value in relation to 1996-98; p-value < 0.05

** p-value in relation to 1996-98; p-value < 0.001

It is important to highlight that the higher decrease in the prevalence of SHS exposure occurred in the period from 1996-98 to 2002-04, but the downward trend was maintained from to 2002-04 to 2008.

### Validity of the biomarker

Table 3 shows that in the first two groups of study there is a good correlation between the number of self-reported smoking parents and cord blood cotinine levels. Clearly, cord blood cotinine level is slightly higher when the father is the only smoker compared when neither parent is a smoker; but when the mother smokes, the increase is much higher. For 2008 the trend is the same, however the differences are smaller without reaching statistical significance.

**Table 3:** Parental smoking habit declared by self-report and probability distribution (mean, median and geometric mean) of cord blood cotinine level

Parental smoking habit				Cord blood cotinine levels (ng/mL)								
	
			1996 98				2002 04				2008	
			N = 373				N = 245				N = 181	
	
	GM	Median	Mean (SD)	p-value	GM	Median Mean (SD)		p-value	GM	Median	Mean (SD)	p-value
Nobody	1.8	1.6	8.4 (57.4)	Ref.	0.4	0.4	0.6 (0.8)	Ref.	0.6	0.37	3.5 (7.9)	Ref.
Only father	3.1	2.4	16.2 (55.5)		0.6	0.5	2.3 (6.4)		0.9	0.31	7.0 (17.3)	
Only mother	62.5	74.6	132.4 (183.1)	**	55.6	75.5	74.9 (50.0)	**	0.7	0.36	9.6 (27.7)	
Both	78.1	97.2	162.0 (170.0)	**	20.8	28.7	39.8 (37.6)	**	0.9	0.29	14.5 (36.1)	

GM: geometric mean; Ref.: reference

** p-value in relation to "Nobody smoke"; p-value < 0.001

To distinguish between newborns from smoking and non-smoking mothers, the cut-off of 14 ng/mL for cord serum cotinine was used. Table 4 shows that cord blood cotinine levels higher than 14 ng/mL correspond to smoking mothers (75.5 - 90.9%), with the exception of 2008 where cord blood cotinine levels are lower for the entire group due to less SHS.

**Table 4:** Parental smoking habit and cotinine levels in cord blood higher 14 ng/mL

Parental smoking habit		Cord blood cotinine levels > 14ng/mL				
	
		1996 98		2002 04		2008
		N = 373		N = 245		N = 181
	
	n	%	n	%	n	%
Nobody	5	3.9 (5/128)	0	0.0 (0/109)	6	7.7 (6/78)
Only father	13	11.8 (13/110)	3	6.9 (3/43)	6	15.0 (6/40)
Only mother	43	86.0 (43/50)	40	90.9 (40/44)	4	14.8 (4/27)
Both	77	90.6 (77/85)	37	75.5 (37/49)	6	16.7 (6/36)

## Discussion

The results show a decreasing trend of exposure to SHS and an increase of prenatal non-exposure to SHS. This improvement was significant before the implementation of Spanish free-smoke legislation in 2005 and maintains the tendency after it. Globally, there was no change in the percentage of women smoking during pregnancy along the years, but there was an increase in the number of non-Spanish women who smoked during pregnancy.

### Prenatal exposure

Our results demonstrate that in recent years the exposure to SHS has decreased as evidenced by a higher percentage of samples with cord blood cotinine < 1 ng/mL; at the same time prenatal exposure to low levels of SHS decreased in recent years (cord blood cotinine in the interval of 1.1 to 14 ng/mL). This reduction in SHS exposure has been observed in England (by questionnaire and cotinine in saliva) [46] and Italy (by questionnaire and cotinine in cord blood) [16,47], The present study adds the objective advantage of serial biomarker determinations.

Moreover, the very high prenatal exposure to SHS (cord blood cotinine > 100 ng/mL) has decreased in all the three groups studied. It is important to point out a more marked decrease in prenatal SHS in 2002-04 immediately before the implementation of Spanish smoke-free legislation.

The growing implication of health care services in smoking cessation, the marked presence of negative messages regarding tobacco in the media and the public dialog prompted by the imminent implementation of smoke-free legislation may have had a cumulative effect on the decreasing trend of smoking prevalence. Following that period, the effect has been maintained due to the implementation of smoke-free legislation and due to the mass media pressure.

Furthermore, our study reasserts the importance of maternal smoking habit during pregnancy. Cotinine levels in newborns are slightly higher when the father smokes compared with the families where neither parent is a smoker. The cotinine levels increase a significantly when the mother is a smoker. In this stage of life, the importance of active maternal smoking is related to the direct passage of cotinine to the foetus across the placenta. This is different from maternal passive SHS when the father smokes; in this case, cotinine arrives to foetus through cord blood, after the mother inhaled the smoke.

### Maternal smoking habit

Cord blood accounts for foetal tobacco exposure during the previous days or hours before collection and not for chronic exposure during the entire gestation. Nevertheless, cord serum cotinine appeared to be the most adequate biomarker of foetal exposure to smoking at the end of pregnancy, distinguishing not only active smoking from passive smoking, but also exposure to ETS from nonexposure [29,33,37,43]. Studying the percentage of women who reported daily smoking during pregnancy, it becomes clear that the data from the self-reported questionnaire (32.8, 25.9 and 34.1%) does not show a significant variation in the patient population between the different periods of study while the percentage of newborns with cord blood cotinine > 14 ng/mL is decreasing progressively (34.2, 23.3 y 14.5%, respectively). The discrepancy between percentages reported by self-reported questionnaire and cord blood cotinine level that identify newborns from smoking mothers could be explained if one takes into consideration that the questionnaire inquires about the entire gestational period while the analytical value of cord blood cotinine refers only to the end of the pregnancy. We believe these data indicate that due to implementation of smoke-free legislation, maternal exposure to SHS in the hours just before delivery has decreased significantly.

The obtained results suggest that cord blood cotinine can be a useful biomarker in population studies monitoring the prevalence of SHS exposure and in preventing a bias related to self-report of active smoking or passive exposure or reluctancy to admit it. Maternal hair would be an adequate reference matrix [43] but it has methodological problems of standardization and accessibility as proven in clinical environment and public health studies.

The strong points of this study are that it is the first study that includes data collected before and after the implementation of Spanish smoke-free law and the prenatal exposure to SHS has been studied objectively by a biomarker measured in a non conventional matrix; it is non invasive for the newborn and prevents the bias inherent to maternal self-reports. Data comparison for different biomarkers in general population during the same period is not available at the moment but it could prove useful in the future. It should be interesting to collect new data after a longer period in order to confirm the stability and the trend in the data obtained.

There may be other factors that have an impact as tobacco control measures: negative messages in mass media campaigns and the public awareness derived from them, the increase in the price of tobacco, and specific public health programs to help smoking pregnant women quit. Nevertheless, our data suggest that the implementation of smoke-free legislation is on the right track in reaching one of its objectives: to "guarantee the non-smokers' rights to breath smoke- free air" as demonstrated by the lower cord blood cotinine level in newborns. However, we can not affirm that it had generated changes to facilitate the "smoking cessation by active smokers". The Cochrane review does not demonstrate a conclusive relationship between media campaigns against smoking in adults and the decrease of tobacco use; the

report about the impact of Spanish Law 28/2005 is pointing in the same direction [48,49]. For this reason, it is necessary to consider other preventivemeasures, as specific actions focused on pregnant women according to the local demographic changes.

## Conclusions

The three study periods (1996-2008) demonstrated a significant increase in the prevalence of newborns free of SHS exposure and a decrease in the prevalence of newborns exposed to SHS during pregnancy, especially at the very high levels of exposure. The change could be explained as a combination of several factors: negative messages in communication media and the public awareness derived from them; the implementation in 2005 of Spanish smoke-free legislation; and the increase in the tobacco price for the fiscal modification.

It is necessary to maintain and intensify the campaigns against prenatal SHS using communication media, more extensive and severe implementation of smoke-free legislation and educational actions in favour of decreasing the smoking habit among pregnant women. All these interventions will make it possible to improve the present results in the future [50].

Our study indicates that there is a significant maternal smoking habit in this geographical area. Our recommendation is that campaigns against smoking should be directed more specifically towards pregnant women with particular emphasis on non-native pregnant smokers due to the highest prevalence of tobacco consumption in the immigrant women.

## Competing interests

The authors declare that they have no competing interests.

## Authors' contributions

CP, analyzed the mother-infant data, reviewed the literature and the final manuscript, and was the main contributor in writing the manuscript. OV, was the paediatrician responsible for coordination of data and samples flow and contributed in writing the manuscript. ÓG-A, analyzed the mother-infant data, reviewed the literature and was a major contributor in writing the manuscript. EP, was an important laboratory technician in biomarkers analyses and contributed in writing the manuscript. SP, was the major expert in laboratory analysis of biomarkers in alternative matrices and contributed in writing the manuscript. ES, analyzed the mother-infant data and was a major contributor in writing the manuscript. JRV, was the expert responsible for final data analysis and contributed in writing the manuscript. All authors read and approved the final manuscript.

## Pre-publication history

The pre-publication history for this paper can be accessed here:

http://www.biomedcentral.com/1471-2393/12/26/prepub
